# Surgical choice of non-small cell lung cancer with unexpected pleural dissemination intraoperatively

**DOI:** 10.1186/s12885-021-08180-1

**Published:** 2021-04-22

**Authors:** Junjie Hu, Yan Chen, Xinsheng Zhu, Qiang Ma, Jing Zhang, Gening Jiang, Peng Zhang

**Affiliations:** Department of Thoracic Surgery, Shanghai Pulmonary Hospital, Tongji University School of Medicine, No. 507 Zhengmin Road, Yangpu District, Shanghai, 200433 P.R. China

**Keywords:** NSCLC, pleural dissemination, surgery, prognosis, adjuvant therapy

## Abstract

**Background:**

Whether patients with non-small cell lung cancer (NSCLC) with unexpected pleural dissemination (UPD) could get survival benefit from tumor resection remained controversial.

**Methods:**

Totally, 169 patients with NSCLC with UPD were included between 2012 and 2016. Patients were divided into the tumor resection and open-close group. Progression-free survival (PFS) and overall survival (OS) were compared with a log-rank test. The multivariable Cox analysis was applied to identify prognostic factors.

**Results:**

Sixty-five patients received open-close surgery and 104 patients underwent main tumor and visible pleural nodule resection. Tumor resection significantly prolonged OS (hazard ratio [HR]: 0.408, *P* < 0.001), local PFS (HR: 0.283, *P* < 0.001), regional PFS (HR: 0.506, *P* = 0.005), and distant metastasis (HR: 0.595, *P* = 0.032). Multivariable Cox analysis confirmed that surgical method was an independent prognostic factor for OS, local PFS and regional PFS, except distant metastasis. Subgroup analyses indicated that tumor resection could not improve OS in the patients who received targeted therapy (HR: 0.649, *P* = 0.382), however, tumor resection was beneficial for the patients who received adjuvant chemotherapy alone (HR: 0.322, *P* < 0.001). In the tumor resection group, lobectomy (HR: 0.960, *P* = 0.917) and systematic lymphadenectomy (HR: 1.512, *P* = 0.259) did not show survival benefit for OS.

**Conclusions:**

Main tumor and visible pleural nodule resection could improve prognosis in patients with UPD who could not receive adjuvant targeted therapy. Sublobar resection without systematic lymphadenectomy may be the optimal procedure.

**Supplementary Information:**

The online version contains supplementary material available at 10.1186/s12885-021-08180-1.

## Introduction

Lung cancer ranked first in terms of the incidence and the mortality among malignant tumors [1], and non-small cell lung cancer (NSCLC) represented approximately 85% of lung cancer cases [2]. Curative surgical resection was the first-line choice for early stage NSCLC, while systematic therapy was the standard of care for advanced NSCLC [3]. Pretreatment evaluation for tumor resectability and metastasis should be conducted before the operation, and methods for evaluation included bronchoscopy, endobronchial ultrasound, positron emission tomography / computer tomography (PET/CT), etc. [3]. Although patients were assessed to clinical stage M0 after evaluations, unexpected pleural dissemination (UPD) was detected occasionally by thoracic surgeons in the operating procedure. The choice of tumor resection or open-close surgery remained controversial.

Recently, several studies revealed that tumor resection could bring survival benefit in the patients with UPD [4–7]. However, the sample size of these studies was small. In addition, these studies did not make subgroup analysis for adjuvant therapy. Given that targeted therapy had a greater survival benefit for advanced NSCLC than conventional chemotherapy [8–10], the survival benefit of tumor resection for the patients who received targeted therapy was unclear. Thus, our aims were to validate the benefit of tumor resection in the patients with UPD and explore its benefit in subgroups of different adjuvant therapeutic regimens.

## Materials and methods

### Study design

The was a retrospective cohort study that was approved by the Ethic Committee of Shanghai Pulmonary Hospital (approved number: K20-283). This analysis was performed in accordance with the Strengthening the Reporting of Cohort Studies in Surgery (STROCSS) criteria [11].

### Patients

We retrospectively reviewed medical records of the consecutive patients who received thoracic surgery between January 2012 and December 2016 in Department of Thoracic Surgery, Shanghai Pulmonary Hospital. The inclusion criteria were: (1) primary pathologic stage IV-M1a NSCLC according to the 8th edition of the TNM staging system [12], (2) clinical stage M0 before the operation, (3) malignant pleural dissemination. The patients were excluded if met any of the following criteria: (1) benign disease, (2) small cell lung cancer, (3) metastatic tumor of other cancer, (4) stage I-III NSCLC, (5) stage IV-M1b or IV-M1c. (6) lost fellow-up (< 3 months).

### Preoperative evaluation

All patients underwent preoperative evaluation for both tumor resectability and metastasis. Bronchoscopy and chest enhanced CT scan were requested for all lung cancer candidates. Distant metastasis was assessed routinely by using brain CT scan or magnetic resonance imaging (MRI), abdominal CT/MRI or sonography and bone scintigraphy. If the patients received PET-CT scan, the examinations above (except bronchoscopy) were not requested. Ultrasonic probing for thoracentesis was performed routinely in patients with preoperative noted pleural effusion, and the drainage liquid was sent for cytology.

### Operations

Video-assisted thoracic surgery (VATS) or standard posterolateral thoracotomy was performed according to the tumor characteristics, and VATS was the first choice generally. Initial exploration was performed and a frozen section of the pleural biopsy was taken if pleural metastasis was suspected. After pathological confirmation of the pleural malignancies, the choice of tumor resection or pleural biopsy alone and the extent of resection depended on surgeons’ (17 chief or deputy chief surgeons) experiences and preferences. If the surgeons did not choose to resect the primary tumor, thorax closure was performed immediately. All the visible pleural lesions of the patients who underwent tumor resection were resected (large lesions) or cauterized by the electrotome (small lesions) as many as possible.

### Adjuvant therapy

Driver gene mutation detection was recommended for all patients. If the patients harboring epidermal growth factor receptor (EGFR) mutation or anaplastic lymphoma kinase (ALK) rearrangement, the corresponding targeted drugs was recommended for first-line treatment. If the driver gene mutations were negative or the patients did not choose a targeted therapy due to cost, allergy, adverse effects or other factors, platinum-based chemotherapy was recommended.

### Follow-up

The patients were scheduled for a first re-visit at 4 weeks after operations, and the follow-up visit was scheduled every 3 - 6 months. Tumor progression events were detected by radiological evaluation (as listed above). As the definitions of previous studies [4, 7], Local progression was defined as the primary lesion enlargement or lesion recurrence at the resection site. Regional progression was defined as increasing pleural effusion / pleural nodules / lung lesions, or ipsilateral lymph node recurrence / enlargement. Distant metastasis was defined as new lesions in the contralateral lung or any other organ (brain, bone, etc.).

### Statistical analysis

Categorical variables were analyzed by the Pearson chi-square test or Fisher’s exact test. Continuous variables were analyzed by the Student’s t test or Wilcoxon rank-sum test. Progression-free survival (PFS) was defined as the time from surgery to any disease progression or the last follow-up. Overall survival (OS) was defined as the time from surgery to death or the last follow-up. Kaplan-Meier method was used to obtain the PFS and OS curves, and a log-rank test was used to compare the curves. Univariable Cox proportional hazard regression was used to identify prognostic factors. Multivariable analysis was performed in the factors with *p* value < 0.10 to identify independent prognostic factors. All analyses were conducted by using R software (version 3.6.3), and a two-sided *P* value of 0.05 was considered statistically significant.

## Results

### Clinicopathological characteristics

**Fig. 1:**
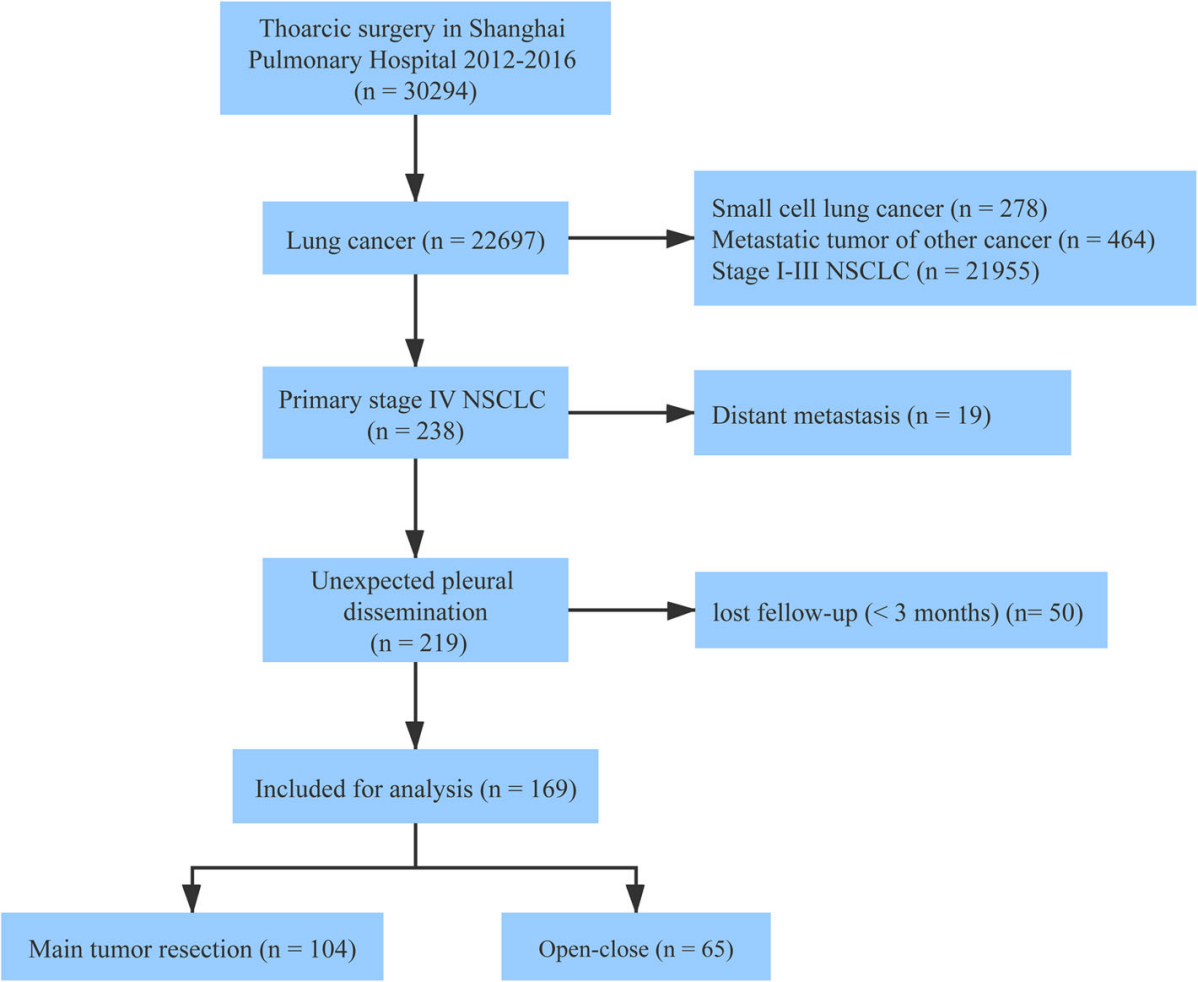
Selection process of the patients in the study

Totally, 169 patients who fulfilled the selection criteria were included in the study **(**Fig. 1). Of the 169 patients, 65 patients received open-close surgery and 104 patients underwent main tumor and visible pleural nodule resection. Table 1 presented the clinical and pathological characteristics in the two groups, and no significant difference was observed. The open-close group included 54 (83.1%) adenocarcinoma, 4 squamous cell carcinoma (SCC), 7 other NSCLC (2 adenosquamous carcinoma, 2 large cell carcinoma, 2 carcinosarcoma and 1 poorly differentiated carcinoma). The tumor resection group included 92 adenocarcinoma, 7 SCC and 5 other NSCLC (3 adenosquamous carcinoma, 1 large cell carcinoma and 1 lymphoepithelioma-like carcinoma). EGFR mutation was detected in 22 and 57 patients in the two groups, respectively. Two cases of ALK rearrangement were detected in the tumor resection group, and one case in the open-close group.

**Table 1:** Clinicopathological characteristics of patients

Variables	Open-close	Tumor resection	*P* value
Number of patients, n	65	104	-
Age, year (mean ± SD)	57.7 ± 9.5	56.9 ± 11.2	0.664
Male gender, n (%)	38 (58.5)	52 (50.0)	0.361
Smoking status, n (%)			0.433
Non-smoker	45 (69.2)	79 (76.0)	
Smoker	20 (30.8)	25 (24.0)	
Comorbidities, n (%)			0.781
Cardiovascular	12 (18.5)	20 (19.2)	
Diabetes	3 (4.6)	8 (7.7)	
Hepatitis	2 (3.1)	0 (0.0)	
Other malignancies	1 (1.5)	1 (1.0)	
Pathological type, n (%)			0.340
Adeno	54 (83.1)	92 (88.5)	
Squamous	4 (6.2)	7 (6.7)	
Other	7 (10.8)	5 (4.8)	
Right-sided tumor, n(%)	33 (50.8)	59 (56.7)	0.550
Clinical T stage, n (%)			0.084
1	27 (41.5)	57 (54.8)	
2	21 (32.3)	35 (33.7)	
3	7 (10.8)	6 (5.8)	
4	10 (15.4)	6 (5.8)	
Clinical N stage, n (%)			0.677
0	36 (55.4)	61 (58.6)	
1	4 (6.1)	9 (8.6)	
2	25 (38.5)	34 (32.7)	
Gene mutation, n (%)			0.007
EGFR/ALK	23 (35.4)	59 (56.7)	
No/Unknown	42 (64.6)	45 (43.3)	
Neoadjuvant treatment, n (%)			
Chemotherapy	2 (3.1)	6 (5.8)	0.668
Targeted therapy	0 (0.0)	1 (1.0)	1.000
Adjuvant treatment, n (%)			
Chemotherapy	49 (75.4)	84 (80.8)	0.523
Targeted therapy	20 (35.1)	44 (45.4)	0.212

*SD* Standard deviation, *EGFR* Epidermal growth factor receptor, *ALK* Anaplastic lymphoma kinase.

### Perioperative outcomes

More patients in the tumor resection group received thoracotomy (35.6% vs 10.8, *P* = 0.001), and the open-close group had a higher proportion of pleural effusion (75.4% vs 23.1%, *P* < 0.001) (Table 2). In the tumor resection group, 67 patients received lobectomy, and 33 patients received sublobar resection (including 2 cases of segmentectomy), and 4 patients received pneumonectomy. Forty-four (42.3%) patients in the tumor resection group underwent systematic lymphadenectomy, while no patients in the open-close group underwent systematic lymphadenectomy. The tumor resection group had significantly longer post-operative hospital stay (6 days vs 4 days, *P* < 0.002). Although the incidence of post-operative complication (19.2%) in the tumor resection group was higher than the open-close group (9.2%), the difference was not significant (*P* = 0.080). There was one case of death in the tumor resection group. The patient was a 57-year-old man, and he suffered from massive pulmonary embolism on the first post-operative day and died 4 days later.

**Table 2:** Perioperative results

Variables	Open-close	Tumor resection	*P* value
Number of patients, n	65	104	-
Approach, n (%)			0.001
Thoracotomy	7 (10.8)	37 (35.6)	
VATS	58 (89.2)	67 (64.4)	
Pleural effusion, n (%)	49 (75.4)	24 (23.1)	< 0.001
Procedure, n (%)			<0.001
Biopsy alone	65 (100.0)	0 (0.0)	
Lobectomy	0 (0.0)	67 (64.4)	
Sublobar resection	0 (0.0)	33^a^ (31.7)	
Pneumonectomy	0 (0.0)	4 (3.8)	
Systematic lymphadenectomy, n(%)	65 (100.0)	60 (57.7)	< 0.001
PHS, day (median [IQR])	4 (3 - 6)	6 (4 -7)	< 0.001
Post-operative complication, n (%)	6 (9.2)	20 (19.2)	0.080
Transfusion	1 (1.5)	8 (7.7)	0.167
SPI	4 (6.1)	9 (8.6)	0.553
Pulmonary thrombosis	0 (0.0)	2 (1.9)	0.524
Prolonged air leak (> 7 days)	0 (0.0)	2 (1.9)	0.524
Chylothorax	0 (0.0)	1 (1.0)	1.000
Atelectasis	1 (1.5)	0 (0.0)	0.385
Mortality, n (%)	0 (0.0)	1 (1.0)	1.000

*VATS* Video-assisted thoracoscopic surgery, *PHS* Postoperative hospital stay, *IQR* Interquartile range, *SPI* Secondary pulmonary infection.

^a^ Two patients received segmentectomy and 31 patients received wedge resection.

### Neoadjuvant and adjuvant therapy

Eight patients (2 in the open-close group and 6 in the tumor resection group) received neoadjuvant chemotherapy (Table 1) and 1 patient underwent targeted therapy followed by chemotherapy. Totally, 126 patients received first-line platinum-based chemotherapy. Of the 65 patients who received targeted therapy, 22 patients received first-line targeted therapy alone, and 23 patients received targeted maintenance therapy followed by chemotherapy, and 20 patients received second-line targeted therapy. Sixty-four patients received first generation tyrosine kinase inhibitors (TKIs) therapy (gefitinib = 43, erlotinib = 13, icotinib = 5, crizotinib = 3), and only one patient received afatinib (second generation TKIs) therapy. Ten patients received third generation TKIs (osimertinib) after drug resistance of first generation TKIs and tumor progression.

### Survival analysis regarding surgical methods

**Fig. 2:**
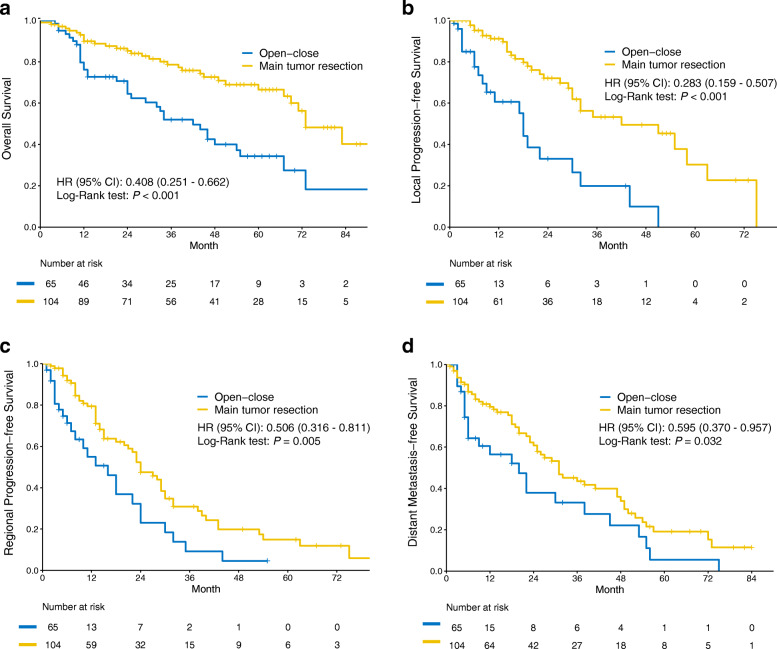
Kaplan–Meier survival curves of the study groups: **a** Overall survival, (**b**) local progression-free survival, (**c**) regional progression-free survival and (**d**) distant metastasis-free survival

The median follow-up time was 24.0 (interquartile range [IQR]: 11.0 - 48.0) months and 39.5 (IQR: 17.2 - 61.2) months in the open-close and tumor resection group, respectively. Kaplan-Meier survival analysis with a log-rank test demonstrated tumor resection could significantly prolong OS (hazard ratio [HR]: 0.408, 95% confidence interval [CI]: 0.251 - 0.662, *P* < 0.001) (Fig. 2). In univariable analysis, besides surgical method, sex, smoking status, clinical T stage, pleural effusion, chemotherapy and targeted therapy were prognostic factors for OS (Table 3). Multivariable Cox analysis confirmed that surgical method was an independent prognostic factor (HR: 0.521, 95% CI: 0.288 - 0.943, *P* = 0.031), and clinical T stage, adjuvant chemotherapy and adjuvant targeted therapy were also independent prognostic factors (Table 3).

**Table 3:** Prognostic factors for overall survival by using the Cox proportional hazard model

Variables	Univariable analysis		Multivariable analysis	
	HR (95% CI)	*P* value	HR (95% CI)	*P* value
Age				
> 65 vs ≤ 65	1.539 (0.904 - 2.619)	0.112	-	-
Sex				
Male vs female	1.982 (1.200 - 3.275)	0.008	1.207 (0.616 - 2.368)	0.582
Smoking status				
Yes vs no	1.713 (1.035 - 2.835)	0.036	1.228 (0.652 - 2.310)	0.524
Comorbidities				
Yes vs no	1.613 (0.930 - 2.799)	0.089	1.580 (0.886 - 2.815)	0.121
Pathological type				
Adeno vs non-adeno	1.879 (0.982 - 3.596)	0.291	1.110 (0.527 - 2.338)	0.783
Clinical T stage				
3-4 vs 1-2	2.841 (1.620 - 4.982)	<0.001	1.959 (1.044 -3.678)	0.036
Clinical N stage				
1-2 vs 0	1.516 (0.936 - 2.455)	0.091	1.010 (0.593 - 1.722)	0.969
Approach				
VATS vs thoracotomy	0.674 (0.401 - 1.134)	0.137	-	-
Pleural effusion				
Yes vs no	2.555 (1.561 - 4.180)	<0.001	1.573 (0.856 - 2.893)	0.144
Surgical method				
Tumor resection vs open-close	0.408 (0.251 - 0.662)	<0.001	0.521 (0.288 - 0.943)	0.031
Neoadjuvant chemotherapy				
Yes vs no	0.908 (0.284 - 2.898)	0.870	-	-
Adjuvant chemotherapy				
Yes vs no	0.612 (0.353 - 1.063)	0.024	0.285 (0.148 - 0.548)	< 0.001
Adjuvant targeted therapy				
Yes vs no	0.249 (0.141 - 0.439)	<0.001	0.193 (0.093 - 0.400)	< 0.001

*HR* Hazard ratio, *CI* Confidence interval, *VATS* Video-assisted thoracoscopic surgery.

As shown in Fig. 2b-d, compared with the open-close group, the tumor resection group had significantly better local PFS (HR: 0.283, 95% CI: 0.159 - 0.507, *P* < 0.001), regional PFS (HR: 0.506, 95% CI: 0.316 - 0.811, *P* = 0.005), and distant metastasis (HR: 0.595, 95% CI: 0.370 - 0.957, *P* = 0.032). Multivariable Cox analysis confirmed that surgical method was an independent prognostic factor for local PFS (Supplementary Table 1) and regional PFS (Supplementary Table 2), however, the result was negative in distant metastasis (Supplementary Table 3).

### Subgroup analysis regarding adjuvant therapy

**Fig. 3:**
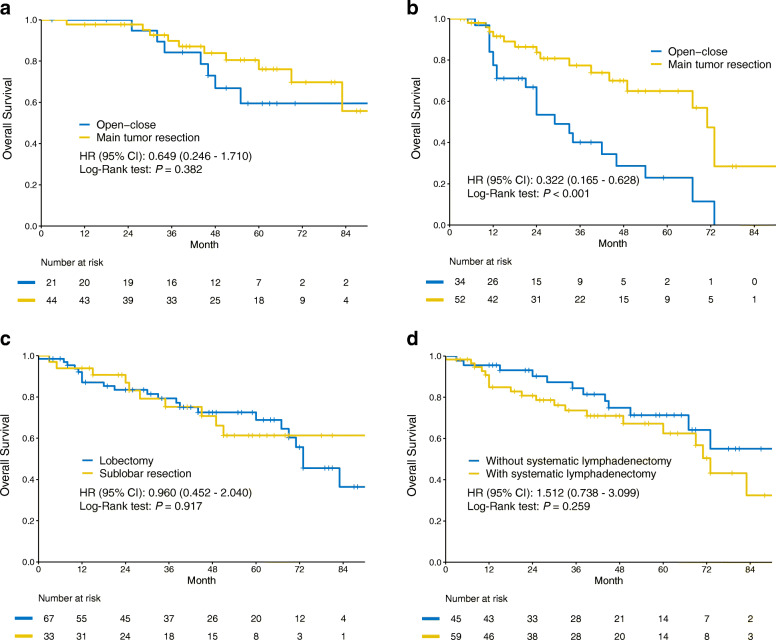
Subgroup analysis of overall survival in the patients who received adjuvant targeted therapy (**a**) and chemotherapy alone (**b**). Subgroup analysis in the tumor resection group regarding surgical extent (**c**) and systematic lymphadenectomy (**d**)

The clinicopathological characteristics of the 65 patients who received targeted therapy were shown in Supplementary Table 4, and they were parallel except higher incidence of pleural effusion in the open-close group (71.4% vs 11.4%, *P* < 0.001), which was coincidence with overall analyses. Kaplan-Meier survival analysis with a log-rank test indicated that tumor resection could not improve OS (HR: 0.649, 95% CI: 0.246 - 1.710, *P* = 0.382) (Fig. 3a). No significant difference was observed among first-line, maintenance therapy after chemotherapy and second-line therapy, and we also observed that administration of third generation TKIs after tumor progression did not significantly improve the OS (Table 4), probably due to small sample size in the subgroups. In multivariable Cox analysis, after adjustment for clinical T stage, N stage, timepoint of TKIs and third generation of TKIs administration, surgical method was still not a risk factor (Table 4).

**Table 4:** Prognostic factors for overall survival of the patients in different therapy groups by using the Cox proportional hazard model

Variables	Targeted therapy				Chemotherapy alone			
	Univariable analysis		Multivariable analysis		Univariable analysis		Multivariable analysis	
	HR (95% CI)	*P* value	HR (95% CI)	*P* value	HR (95% CI)	*P* value	HR (95% CI)	*P* value
Sex								
Male vs female	1.947 (0.746 - 5.082)	0.173	-	-	1.367 (0.673 - 2.776)	0.388	-	-
Smoking status								
Yes vs no	1.677 (0.586 - 4.804)	0.335	-	-	1.519 (0.776 - 2.972)	0.223	-	-
Comorbidities								
Yes vs no	1.018 (0.292 - 3.544)	0.978	-	-	1.557 (0.733 - 3.310)	0.250	-	-
Clinical T stage								
3-4 vs 1-2	1.842 (0.418 - 8.116)	0.420	1.214 (0.231 - 6.365)	0.819	2.100 (0.995 - 4.430)	0.051	1.921 (1.031 - 3.581)	0.040
Clinical N stage								
1-2 vs 0	1.668 (0.631 - 4.410)	0.302	1.458 (0.488 - 4.353)	0.500	1.234 (0.646 - 2.357)	0.524	1.312 (0.777 - 2.213)	0.309
Pleural effusion								
Yes vs no	1.717 (0.627 - 4.704)	0.293	-	-	2.219 (1.134 - 4.341)	0.020	1.538 (0.847 - 2.793)	0.157
Surgical method								
Tumor resection vs open-close	0.649 (0.246 - 1.710)	0.382	0.684 (0.240 - 1.946)	0.476	0.322 (0.165 - 0.628)	< 0.001	0.546 (0.298 - 0.999)	0.049
Time point of TKIs								
First-line	1.000	-	1.000	-	-	-	-	-
Maintenance after chemotherapy	1.482 (0.483 - 4.554)	0.492	1.354 (0.434 - 4.623)	0.601	-	-	-	-
Second-line	0.978 (0.260 - 3.678)	0.974	1.164 (0.293 - 4.623)	0.829	-	-	-	-
III generation TKIs								
Yes vs no	0.659 (0.148 - 2.927)	0.584	0.728 (0.153 - 3.467)	0.690	-	-	-	-

*HR* Hazard ratio, *CI* Confidence interval, *TKIs* Tyrosine kinase inhibitors.

In the 78 patients (Supplementary Table 4) who received adjuvant chemotherapy alone, tumor resection could significantly prolong OS (HR: 0.322, 95% CI: 0.165 - 0.628, *P* < 0.001) (Fig. 3b), and it remained positive in multivariable analysis adjusted for clinical T stage, N stage and pleural effusion (Table 4)

### Subgroup analysis in the tumor resection group

The 4 patients underwent pneumonectomy were excluded in the subgroup analysis, and 67 patients underwent lobectomy and 33 patients underwent sublobar resection. The baseline characteristics of the two groups were similar, especially for clinical T and N stage (Supplementary Table 5). We noticed that more patients in the lobectomy group underwent systematic lymphadenectomy (80.6% vs 6.1%, *P* < 0.001). The Kaplan-Meier survival analysis with a log-rank test showed that there was no significant difference between lobectomy and sublobar resection for OS (HR: 0.960, 95% CI: 0.452 - 2.040, *P* = 0.917) (Fig. 3c), and it was also negative in multivariable analysis (Supplementary Table 6) We also observed that systematic lymphadenectomy could not improve OS in Kaplan-Meier plot (HR: 1.512, 95% CI: 0.738 - 3.099, *P* = 0.259, Fig. 3d) and Cox analysis (Supplementary Table 6), with the similar clinical N stage (Supplementary Table 5). Given the potential interaction between resection extent and systematic lymphadenectomy, we also analyzed systematic lymphadenectomy in the patients who underwent lobectomy, and it remained negative (Supplementary Figure 1).

## Discussion

NSCLC with pleural or pericardial dissemination was categorized into stage IV-M1a in the 7th and 8th TNM staging system [12, 13], which was generally not recommended for surgery, according to the National Comprehensive Cancer Network (NCCN) guidelines [3]. However, UPD was occasionally met in the operation procedure by surgeons, and surgeons chose the surgical method according to their experiences and preferences. In the study, significantly higher rate of pleural effusion in the open-close group was observed, which indicated that surgeons may prefer to open-close surgery due to pleural effusion. However, the multivariable analysis showed that pleural effusion was not independent prognostic factors for OS. Li et al [4] found that clinical T stage was higher in the open-close group than the tumor resection group, which may associate with a surgeon’s tendency to select open-close procedures.

In this retrospective study, we observed that tumor resection had better OS than open-close surgery in 169 patients with UPD, which was in concordance with previous studies [4–7]. Ren et al [5] reported 83 cases in our center from 2005 to 2013, and they found that primary tumor resection had significantly better OS compared with biopsy in patients with UPD (3-year OS, 45.8% vs 11.8%, *P* = 0.001). They also analyzed the survival data of patients with ipsilateral pleural effusion (stage M1a) from the Surveillance, Epidemiology, and End Results database, and they also observed the similar result (HR: 2.58, 95% CI: 1.84 - 3.61, *P* < 0.001) [6]. Li et al [4] analyzed 43 patients with lung adenocarcinoma with intraoperatively diagnosed pleural seeding, and A significantly higher 3-year OS was observed in the tumor resection group than open-close surgery (82.9% vs 38.5%, *P* = 013). The results from Yun and colleagues’ study in 78 patients localized pleural seeding demonstrated that tumor resection could increase 3-year survival rate (66.7% vs 41.1%, *P* = 0.012). A meta-analysis including 9 studies also concluded that tumor resection had significant survival benefit (HR: 0.443, 95% CI: 0.344 - 0.571, *P* < 0.001) [14]. NSCLC with pleural or pericardial dissemination was generally not recommended for surgery [3], and the consensus favored open-close surgery followed by chemotherapy or targeted therapy for stage IV disease [15]. However, these studies indicated that tumor resection could be an option in multimodality treatment. Besides advanced disease, surgery associated complication was another concern for tumor resection. In our study, we did not observe a significantly higher incidence of post-operative complication in the tumor resection group, and Ren et al [5] and Yun et al [7] also reported the same result.

We observed better local and regional PFS in the tumor resection group, which was consistent with previous studies reported by Li et al [4] and Yun et al [7] . Tumor resection significantly reduced tumor volume, and larger volume was associated with poor local control [16]. Miura et al [17] claimed that pleural seeding originates from direct or local extension of the tumor via the subpleural lymphatic system. In terms of distant metastasis, the positive result in the Kaplan-Meier survival curves did not be confirmed by multivariable Cox analysis. Li et al [4] and Yun et al [7] also found that tumor resection could not improve distant metastasis-free survival.

Targeted therapy recommended by the NCCN guidelines was the first-line therapy for advanced NSCLC harboring EGFR mutation or ALK rearrangement [3]. The greater response rate and survival benefit of targeted therapy than chemotherapy had been validated by several large phase 3 clinical trials [18–23]. Thus, we thought that targeted therapy may affect the benefit of tumor resection. In subgroup analysis, we found that patients could not get survival benefit from tumor resection if they received targeted therapy, while tumor resection could improve OS in the patients who received chemotherapy alone. The results was in accordance with the recent study that reported by Li et al [24]. These indicated that tumor resection may be only beneficial for a subgroup of patients with UPD who did not have the driver gene mutation or could not receive targeted therapy due to cost, allergy, adverse events or other factors. However, the result of diver gene detection should be available for thoracic surgeons when making decision.

The surgical extent had been analyzed in previous studies [4, 7, 25, 26], and they concluded that compared with sublobar resection, lobectomy could not improve prognosis for stage M1a NSCLC. In our study, we also got the same result in subgroup analysis. In addition, we also analyzed the effect of systematic lymphadenectomy in the tumor resection group, and the result demonstrated that systematic lymphadenectomy could not bring survival benefit. These results were not surprising, because tumor resection was a debulking surgery rather than curative surgery for the patients with stage M1a NSCLC.

There were several limitations in our study. First, some biases were inevitable because of the retrospective and single-center nature of this study. Selection bias probably existed in the choice of surgical method, and higher incidence of pleural effusion was observed in the open-close group, which may be associated with a surgeon’s tendency to select open-close surgery. Second, the sample size was not big enough, especially for subgroup analyses, although it was the largest one among the recent studies.

## Conclusions

This study indicated that main tumor and visible pleural nodule resection could improve OS and PFS for the patients with UPD, especially for the patients who could not receive adjuvant targeted therapy. For the patients harboring driver gene mutations, tumor resection may be not beneficial for prognosis due to the great benefit of targeted therapy. Sublobar resection without systematic lymphadenectomy may be the optimal procedure, because extensive resection and systematic lymphadenectomy could not improve prognosis. Large-scale, prospective studies were warranted to validate the benefit of tumor resection for stage M1a NSCLC.

## Supplementary Information

**Additional file 1: Table** **1.** Prognostic factors for local progression-free survival by using the Cox proportional hazard model. **Table 2.** Prognostic factors for regional progression-free survival by using the Cox proportional hazard model. **Table 3.** Prognostic factors for distant metastasis-free survival by using the Cox proportional hazard model. **Table 4.** Clinicopathological characteristics of patients in different therapy subgroups. **Table 5**. Clinicopathological characteristics of patients who underwent tumor resection. **Table 6**. Prognostic factors for overall survival of the patients who underwent tumor resection by using the Cox proportional hazard model. **Figure 1.** Subgroup analysis in the lobectomy group regarding systematic.

## Data Availability

The raw data of this study are derived from our hospital. All detailed data included in the study are available upon request by contact with the corresponding author.
