# Supplementary material for: Surgical choice of non-small cell lung cancer with unexpected pleural dissemination intraoperatively
Source: BMC Cancer. 2021 Apr 22;21:445. doi: 10.1186/s12885-021-08180-1 (PMC8061008; doi:10.1186/s12885-021-08180-1)
Supplement: Supplementary file 1 — Additional file 1: Table 1. Prognostic factors for local progression-free survival by using the Cox proportional hazard model. Table 2. Prognostic factors for regional progression-free survival by using the Cox proportional hazard model. Table 3. Prognostic factors for distant metastasis-free survival by using the Cox proportional hazard model. Table 4. Clinicopathological characteristics of patients in different therapy subgroups. Table 5. Clinicopathological characteristics of patients who underwent tumor resection. Table 6. Prognostic factors for overall survival of the patients who underwent tumor resection by using the Cox proportional hazard model. Figure 1. Subgroup analysis in the lobectomy group regarding systematic. [file 12885_2021_8180_MOESM1_ESM.docx]

**Supplementary Table**

Table 1. Prognostic factors for local progression-free survival by using the Cox proportional hazard model

| Variables | | Univariable analysis | | Multivariable analysis | |
| --- | --- | --- | --- | --- | --- |
|  |  | HR (95% CI) | P value | HR (95% CI) | P value |
| Age | |  |  |  |  |
|  | > 65 vs ≤ 65 | 0.875 (0.422 - 1.815) | 0.721 | - | - |
| Sex | |  |  |  |  |
|  | Male vs female | 1.260 (0.723 - 2.197) | 0.415 | - | - |
| Smoking status | |  |  |  |  |
|  | Yes vs no | 1.057 (0.552 - 2.025) | 0.866 | - | - |
| Comorbidities | |  |  |  |  |
|  | Yes vs no | 0.753 (0.339 - 1.675) | 0.487 | - | - |
| Pathological type | |  |  |  |  |
|  | Adeno vs non-adeno | 0.628 (0.672 - 3.776) | 0.835 | - | - |
| Clinical T stage | |  |  |  |  |
|  | 3-4 vs 1-2 | 0.665 (0.238 - 1.853) | 0.435 | - | - |
| Clinical N stage | |  |  |  |  |
|  | 1-2 vs 0 | 1.040 (0.587 - 1.841) | 0.893 | - | - |
| Approach | |  |  |  |  |
|  | VATS vs thoracotomy | 2.286 (1.071 - 4.880) | 0.032 | 2.015 (0.902 - 4.503) | 0.087 |
| Pleural effusion | |  |  |  |  |
|  | Yes vs no | 2.382 (1.292 - 4.391) | 0.005 | 1.299 (0.669 - 2.520) | 0.439 |
| Surgical method | |  |  |  |  |
|  | Tumor resection vs open-close | 0.284 (0.159 - 0.507) | < 0.001 | 0.340 (0.177 - 0.655) | 0.001 |
| Neoadjuvant chemotherapy | |  |  |  |  |
|  | Yes vs no | 1.594 (0.567 - 4.481) | 0.376 | - | - |
| Adjuvant chemotherapy | |  |  |  |  |
|  | Yes vs no | 0.256 (0.078 - 0.838) | 0.024 | 0.299 (0.084 - 1.068) | 0.063 |
| Adjuvant targeted therapy | |  |  |  |  |
|  | Yes vs no | 0.599 (0.329 - 1.091) | 0.094 | 0.567 (0.298 - 1.081) | 0.085 |

HR: hazard ratio, CI: confidence interval.

Table 2. Prognostic factors for regional progression-free survival by using the Cox proportional hazard model

| Variables | | Univariable analysis | | Multivariable analysis | |
| --- | --- | --- | --- | --- | --- |
|  |  | HR (95% CI) | P value | HR (95% CI) | P value |
| Age | |  |  |  |  |
|  | > 65 vs ≤ 65 | 0.884 (0.509 - 1.535) | 0.661 | - | - |
| Sex | |  |  |  |  |
|  | Male vs female | 1.566 (1.010 - 2.428) | 0.045 | 1.498 (0.964 - 2.326) | 0.072 |
| Smoking status | |  |  |  |  |
|  | Yes vs no | 1.091 (0.655 - 1.818) | 0.738 | - | - |
| Comorbidities | |  |  |  |  |
|  | Yes vs no | 1.058 (0.609 - 1.836) | 0.842 | - | - |
| Pathological type | |  |  |  |  |
|  | Adeno vs non-adeno | 0.928 (0.400 - 2.153) | 0.863 | - | - |
| Clinical T stage | |  |  |  |  |
|  | 3-4 vs 1-2 | 0.965 (0.481 - 1.933) | 0.919 | - | - |
| Clinical N stage | |  |  |  |  |
|  | 1-2 vs 0 | 0.879 (0.559 - 1.382) | 0.577 | - | - |
| Approach | |  |  |  |  |
|  | VATS vs thoracotomy | 1.246 (0.736 - 2.110) | 0.412 | - | - |
| Pleural effusion | |  |  |  |  |
|  | Yes vs no | 1.731 (1.069 - 2.802) | 0.026 | 1.482 (0.897 - 2.449) | 0.125 |
| Surgical method | |  |  |  |  |
|  | Tumor resection vs open-close | 0.506 (0.316 - 0.811) | 0.005 | 0.583 (0.356 - 0.955) | 0.032 |
| Neoadjuvant chemotherapy | |  |  |  |  |
|  | Yes vs no | 0.949 (0.346 - 2.600) | 0.919 | - | - |
| Adjuvant chemotherapy | |  |  |  |  |
|  | Yes vs no | 1.436 (0.737 - 2.797) | 0.288 | - | - |
| Adjuvant targeted therapy | |  |  |  |  |
|  | Yes vs no | 0.727 (0.462 - 1.143) | 0.167 | - | - |

HR: hazard ratio, CI: confidence interval.

Table 3. Prognostic factors for distant metastasis-free survival by using the Cox proportional hazard model

| Variables | | Univariable analysis | | Multivariable analysis | |
| --- | --- | --- | --- | --- | --- |
|  |  | HR (95% CI) | P value | HR (95% CI) | P value |
| Age | |  |  |  |  |
|  | > 65 vs ≤ 65 | 0.977 (0.565 - 1.689) | 0.935 | - | - |
| Sex | |  |  |  |  |
|  | Male vs female | 1.525 (0.978 - 2.379) | 0.063 | 1.245 (0.755 - 2.053) | 0.389 |
| Smoking status | |  |  |  |  |
|  | Yes vs no | 1.175 (0.708 - 1.950) | 0.533 | - | - |
| Comorbidities | |  |  |  |  |
|  | Yes vs no | 1.106 (0.617 - 1.982) | 0.735 | - | - |
| Pathological type | |  |  |  |  |
|  | Adeno vs non-adeno | 0.480 0.253 - 0.912) | 0.025 | 0.589 (0.291 - 1.192) | 0.141 |
| Clinical T stage | |  |  |  |  |
|  | 3-4 vs 1-2 | 2.057 (1.101 - 3.843) | 0.024 | 1.325 (0.644 - 2.726) | 0.445 |
| Clinical N stage | |  |  |  |  |
|  | 1-2 vs 0 | 1.346 (0.861 - 2.103) | 0.192 | - | - |
| Approach | |  |  |  |  |
|  | VATS vs thoracotomy | 0.885 (0.538 - 1.457) | 0.632 | - | - |
| Pleural effusion | |  |  |  |  |
|  | Yes vs no | 1.638 (1.034 - 2.594) | 0.035 | 1.179 (0.697 - 1.995) | 0.539 |
| Surgical method | |  |  |  |  |
|  | Tumor resection vs open-close | 0.595 (0.370 - 0.957) | 0.032 | 0.572 (0.323 - 1.012) | 0.055 |
| Neoadjuvant chemotherapy | |  |  |  |  |
|  | Yes vs no | 1.151 (0.361 - 3.670) | 0.812 | - | - |
| Adjuvant chemotherapy | |  |  |  |  |
|  | Yes vs no | 0.435 (0.209 - 0.905) | 0.026 | 0.545 (0.253 - 1.173) | 0.121 |
| Adjuvant targeted therapy | |  |  |  |  |
|  | Yes vs no | 0.478 (0.301 - 0.762) | 0.002 | 0.575 (0.328 - 1.011) | 0.055 |

HR: hazard ratio, CI: confidence interval.

Table 4. Clinicopathological characteristics of patients in different therapy subgroups.

| Variables | | Targeted therapy | | | Chemotherapy alone | | |
| --- | --- | --- | --- | --- | --- | --- | --- |
|  |  | Open-close | Tumor resection | P value | Open-close | Tumor resection | P value |
| Number of patients, n | | 21 | 44 | - | 34 | 52 | - |
| Age, year (mean ± SD) | | 58.8 ± 8.9 | 56.7 ± 10.1 | 0.424 | 55.5 ± 9.1 | 56.2 ± 12.1 | 0.758 |
| Male gender, n (%) | | 8 (38.1) | 14 (31.8) | 0.826 | 23 (67.6) | 33 (63.5) | 0.868 |
| Smoking status, n (%) | |  |  | 0.988 |  |  |  |
|  | Non-smoker | 17 (81.0) | 34 (77.3) |  | 21 (61.8) | 40 (76.9) | 0.204 |
|  | Smoker | 4 (19.0) | 10 (22.7) |  | 13 (38.2) | 12 (23.1) |  |
| Comorbidities, n (%) | |  |  | 0.503 |  |  | 0.591 |
|  | Cardiovascular | 5 (23.8) | 6 (13.6) |  | 3 (8.8) | 11 (21.1) |  |
|  | Diabetes | 2 (9.5) | 3 (6.8) |  | 1 (2.9) | 5 (9.6) |  |
|  | Hepatitis | 0 (0.0) | 0 (0.0) |  | 2 (5.8) | 0 (0.0) |  |
|  | Other malignancies | 0 (0.0) | 0 (0.0) |  | 0 (0.0) | 1 (1.9) |  |
| Pathological type, n (%) | |  |  | - |  |  | 0.731 |
|  | Adeno | 21 (100.0) | 44 (100.0) |  | 29 (85.3) | 44 (84.6) |  |
|  | Squamous | 0 (0.0) | 0 (0.0) |  | 2 (5.9) | 5 (9.6) |  |
|  | Other | 0 (0.0) | 0 (0.0) |  | 3 (8.8) | 3 (5.8) |  |
| Right-sided tumor, n (%) | | 11 (52.4) | 27 (61.4) | 0.676 | 15 (44.1) | 27 (51.9) | 0.626 |
| Clinical T stage, n (%) | |  |  | 0.175 |  |  | 0.521 |
|  | 1 | 10 (47.6) | 27 (61.4) |  | 14 (41.2) | 26 (50.0) |  |
|  | 2 | 8 (38.1) | 16 (36.4) |  | 10 (29.4) | 17 (32.7) |  |
|  | 3 | 1 (4.8) | 1 (2.3) |  | 6 (17.6) | 4 (7.7) |  |
|  | 4 | 2 (9.5) | 0 (0.0) |  | 4 (11.8) | 5 (9.6) |  |
| Clinical N stage, n (%) | |  |  | 0.482 |  |  | 0.934 |
|  | 0 | 13 (61.9) | 31 (70.5) |  | 18 (52.9) | 26 (50.0) |  |
|  | 1 | 1 (4.8) | 4 (9.1) |  | 2 (5.9) | 4 (7.7) |  |
|  | 2 | 7 (33.3) | 9 (20.5) |  | 14 (41.2) | 22 (42.3) |  |
| Gene mutation, n (%) | |  |  | 0.953 |  |  |  |
|  | EGFR | 20 (95.2) | 42 (95.5) |  |  |  |  |
|  | ALK | 1 (4.8) | 2 (4.5) |  |  |  |  |
| Approach | |  |  | 0.051 |  |  | 0.022 |
|  | VATS | 20 (95.2) | 31 (70.5) |  | 30 (88.2) | 33 (63.5) |  |
|  | Thoracotomy |  |  |  | 4 (11.8) | 19 (36.5) |  |
| Pleural effusion, n (%) | | 15 (71.4) | 5 (11.4) | < 0.001 | 27 (79.4) | 16 (30.8) | < 0.001 |
| TKIs, n (%) | |  |  |  |  |  |  |
|  | I | 21 (100.0) | 43 (97.7) | 1.000 | - | - | - |
|  | II | 0 (0) | 1 (2.3) | 1.000 | - | - | - |
|  | III* | 3 (14.3) | 7 (15.9) | 0.883 | - | - | - |
| Time point of TKIs, n (%) | |  |  | 0.698 |  |  | - |
|  | First line | 6 (31.6) | 14 (31.8) |  | - | - | - |
|  | Maintenance after chemotherapy | 8 (42.1) | 15 (34.1) |  | - | - | - |
|  | Second line | 5 (23.8) | 15 (34.1) |  | - | - | - |

SD: Standard deviation, EGFR: epidermal growth factor receptor, ALK: anaplastic lymphoma kinase, TKIs: tyrosine kinase inhibitors.

* All the ten patients received III generation TKIs after drug resistance of I generation TKIs and tumor progression.

Table 5. Clinicopathological characteristics of patients who underwent tumor resection.

|  | | Resection extent | | | Systematic lymphadenectomy | | |
| --- | --- | --- | --- | --- | --- | --- | --- |
| Variables | | Lobectomy | Sublobar resection | P value | With | Without | P value |
| Number of patients, n | | 67 | 33 | - | 59 | 45 |  |
| Age, year (mean ± SD) | | 58.1 ± 10.3 | 53.5 ± 12.4 | 0.052 | 57.6 ± 11.4 | 56.0 ± 11.0 | 0.473 |
| Male gender, n (%) | | 35 (52.2) | 13 (39.4) | 0.319 | 36 (61.0) | 16 (35.6) | 0.018 |
| Smoking status, n (%) | |  |  | 0.028 |  |  | 0.003 |
|  | Non-smoker | 46 (68.7) | 30 (90.9) |  | 38 (64.4) | 41 91.1) |  |
|  | Smoker | 21 (31.3) | 3 (9.1) |  | 21 (35.6) | 4 (8.9) |  |
| Comorbidities, n (%) | |  |  | 0.156 |  |  | 0.328 |
|  | Cardiovascular | 16 (23.8) | 4 (12.1) |  | 13 (22.0) | 7 (15.5) |  |
|  | Diabetes | 6 (8.9) | 2 (6.0) |  | 6 (10.1) | 2 (4.4) |  |
|  | Hepatitis | 0 (0.0) | 0 (0.0) |  | 0 (0.0) | 0 (0.0) |  |
|  | Other malignancies | 1 (1.5) | 0 (0.0) |  | 1 (1.7) | 0 (0.0) |  |
| Pathological type, n (%) | |  |  | 0.882 |  |  | 0.988 |
|  | Adeno | 61 (91.0) | 31 (93.9) |  | 52 (88.1) | 40 (88.9) |  |
|  | Squamous | 3 (4.5) | 1 (3.0) |  | 3 (5.1) | 2 (4.4) |  |
|  | Other | 3 (4.5) | 1 (3.0) |  | 4 (6.8) | 3 (6.7) |  |
| Right-sided tumor, n (%) | | 42 (62.7) | 16 (48.5) | 0.255 | 35 (59.3) | 24 (53.3) | 0.681 |
| Clinical T stage, n (%) | |  |  | 0.221 |  |  | 0.235 |
|  | 1 | 34 (50.7) | 23 (69.7) |  | 29 (49.2) | 28 (62.2) |  |
|  | 2 | 25 (37.3) | 9 (27.3) |  | 20 (33.9) | 15 (33.3) |  |
|  | 3 | 4 (6.0) | 1 (3.0) |  | 5 (8.5) | 1 (2.2) |  |
|  | 4 | 4 (6.0) | 0 (0.0) |  | 5 (8.5) | 1 (2.2) |  |
| Clinical N stage, n (%) | |  |  | 0.546 |  |  | 0.241 |
|  | 0 | 37 (55.2) | 22 (66.7) |  | 31 (52.5) | 30 (66.7) |  |
|  | 1 | 6 (9.0) | 2 (6.1) |  | 7 (11.9) | 2 (4.4) |  |
|  | 2 | 24 (35.8) | 9 (27.3) |  | 21 (35.6) | 13 (28.9) |  |
| Gene mutation, n (%) | |  |  | 0.468 |  |  | 0.074 |
|  | EGFR/ALK | 36 (53.7) | 21 (63.6) |  | 20 (33.9) | 24 (53.3) |  |
|  | No/Unknown | 31 (46.3) | 12 (36.4) |  | 39 (66.1) | 21 (46.7) |  |
| Systematic lymphadenectomy, n (%) | | 54 (80.6) | 2 (6.1) | <0.001 | 59 (100.0) | 0 (0.0) | - |
| Pleural effusion, n (%) | | 12 (17.9) | 10 (30.3) | 0.250 | 12 (20.3) | 12 (26.7) | 0.600 |
| Procedure, n (%) | |  |  | - |  |  | < 0.001 |
|  | Lobectomy | 67 (0.0) | 0 (0.0) |  | 54 (91.5) | 13 (28.9) |  |
|  | Sublobar resection | 0 (0.0) | 33 (100.0) |  | 3 (5.1) | 1 (2.2) |  |
|  | Pneumonectomy | 0 (0.0) | 0 (0.0) |  | 2 (3.4) | 31 (68.9) |  |

SD: Standard deviation, EGFR: epidermal growth factor receptor, ALK: anaplastic lymphoma kinase

Table 6. Prognostic factors for overall survival of the patients who underwent tumor resection by using the Cox proportional hazard model

| Variables | | Surgical extent | | | | Systematic lymphadenectomy | | | |
| --- | --- | --- | --- | --- | --- | --- | --- | --- | --- |
|  |  | Univariable analysis | | Multivariable analysis | | Univariable analysis | | Multivariable analysis | |
|  |  | HR (95% CI) | P value | HR (95% CI) | P value | HR (95% CI) | P value | HR (95% CI) | P value |
| Sex | |  |  |  |  |  |  |  |  |
|  | Male vs female | 1.736 (0.856 - 3.520) | 0.127 | - | - | 1.658 (0.817 - 3.364) | 0.161 | - | - |
| Smoking status | |  |  |  |  |  |  |  |  |
|  | Yes vs no | 1.131 (0.517 - 2.473) | 0.758 | - | - | 1.126 (0.515 - 2.462) | 0.766 | - | - |
| Comorbidities | |  |  |  |  |  |  |  |  |
|  | Yes vs no | 2.145 (0.985 - 4.669) | 0.055 | 2.229 (0.949 - 5.235) | 0.065 | 2.202 (1.012 - 4.794) | 0.047 | 2.338 (1.034 - 5.286) | 0.041 |
| Pathological type | |  |  |  |  |  |  |  |  |
|  | Adeno vs non-adeno | 2.040 (0.712 - 5.849) | 0.184 | - | - | 1.540 (0.537 - 4.414) | 0.421 | - |  |
| Clinical T stage | |  |  |  |  |  |  |  |  |
|  | 3-4 vs 1-2 | 3.396 (1.280 - 9.006) | 0.014 | 2.871 (0.989 - 8.337) | 0.052 | 2.849 (1.072 - 7.571) | 0.036 | 1.937 (0.647 - 5.802) | 0.237 |
| Clinical N stage | |  |  |  |  |  |  |  |  |
|  | 1-2 vs 0 | 1.187 (0.585 - 2.407) | 0.635 | 0.846 (0.395 - 1.813) | 0.669 | 1.195 (0.589 - 2.425) | 0.621 | 0.763 (0.351 - 1.661) | 0.496 |
| Pleural effusion | |  |  |  |  |  |  |  |  |
|  | Yes vs no | 2.936 (1.356 - 6.357) | 0.006 | 2.235 (0.921 - 5.425) | 0.075 | 2.805 (1.291 - 6.095) | 0.009 | 2.329 (0.964 - 5.624) | 0.060 |
| Procedure | |  |  |  |  |  |  |  |  |
|  | Lobectomy vs sublobar | 0.960 (0.452 - 2.040) | 0.917 | 0.997 (0.415 - 2.396) | 0.994 | 1.041 (0.452 - 2.040) | 0.917 | - | - |
| Systematic lymphadenectomy | |  |  |  |  |  |  |  |  |
|  | Yes vs no | 1.511 (0.737 - 3.097) | 0.259 |  |  | 1.512 (0.737 - 3.099) | 0.259 | 1.597 (0.706 - 3.613) | 0.261 |
| Neoadjuvant chemotherapy | |  |  |  |  |  |  |  |  |
|  | Yes vs no | 1.180 (0.280 - 4.976) | 0.821 | - | - | 1.212 (0.287 - 5.107) | 0.794 | - | - |
| Adjuvant chemotherapy | |  |  |  |  |  |  |  |  |
|  | Yes vs no | 0.804 (0.347 - 1.861) | 0.610 | - | - | 0.783 (0.338 - 1.815) | 0.570 | - | - |
| Adjuvant targeted therapy | |  |  |  |  |  |  |  |  |
|  | Yes vs no | 0.327 (0.150 - 0.709) | 0.005 | 0.423 (0.186 - 0.965) | 0.041 | 0.335 (0.154 - 0.727) | 0.006 | 0.435 (0.190 - 0.995) | 0.049 |

HR: hazard ratio, CI: confidence interval.

**Supplementary Figure**


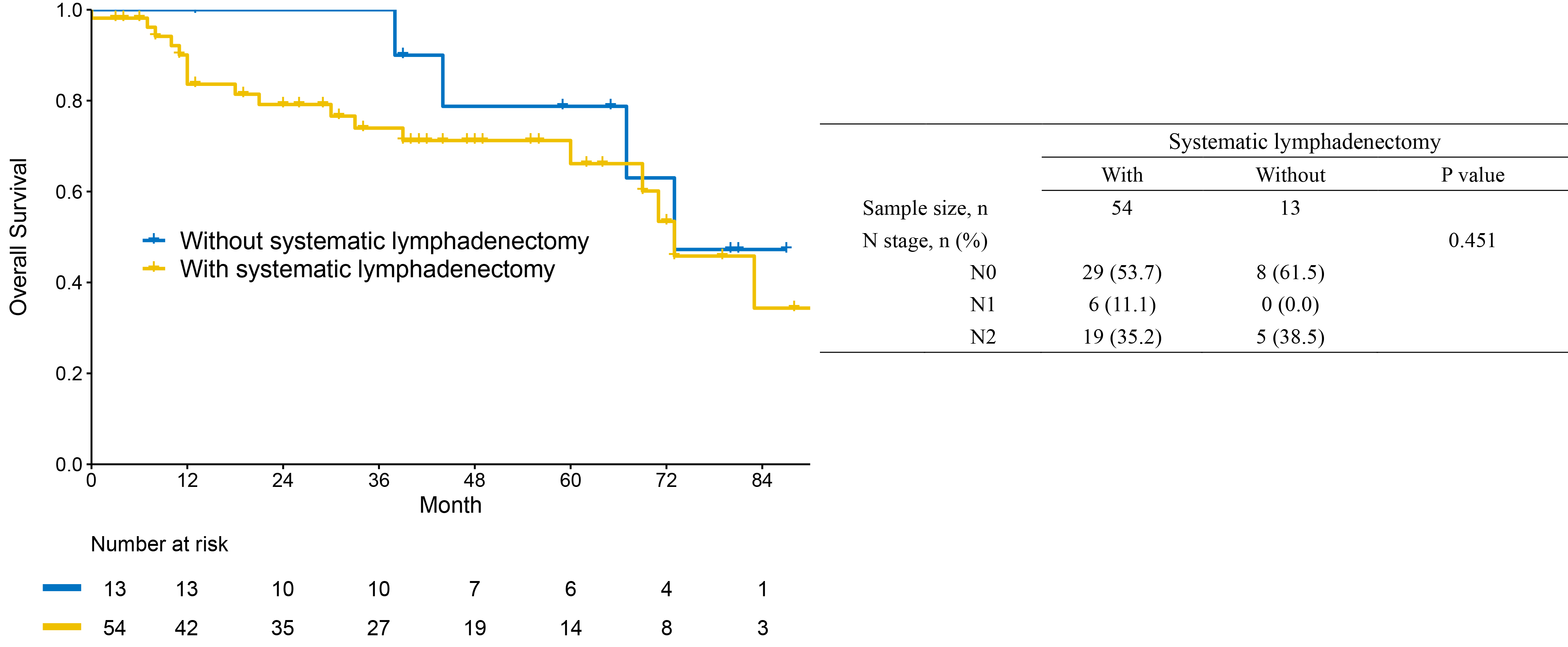


**Figure 1.** Subgroup analysis in the lobectomy group regarding systematic lymphadenectomy.
